# Learning to Choose: Behavioral Dynamics Underlying the Initial Acquisition of Decision-Making

**DOI:** 10.1523/ENEURO.0142-24.2024

**Published:** 2024-05-17

**Authors:** Samantha R. White, Michael W. Preston, Kyra Swanson, Mark Laubach

**Affiliations:** Department of Neuroscience, American University, Washington, DC 20016

**Keywords:** choice, drift diffusion, rat, response time, vision

## Abstract

Current theories of decision-making propose that decisions arise through competition between choice options. Computational models of the decision process estimate how quickly information about choice options is integrated and how much information is needed to trigger a choice. Experiments using this approach typically report data from well-trained participants. As such, we do not know how the decision process evolves as a decision-making task is learned for the first time. To address this gap, we used a behavioral design separating learning the value of choice options from learning to make choices. We trained male rats to respond to single visual stimuli with different reward values. Then, we trained them to make choices between pairs of stimuli. Initially, the rats responded more slowly when presented with choices. However, as they gained experience in making choices, this slowing reduced. Response slowing on choice trials persisted throughout the testing period. We found that it was specifically associated with increased exponential variability when the rats chose the higher value stimulus. Additionally, our analysis using drift diffusion modeling revealed that the rats required less information to make choices over time. These reductions in the decision threshold occurred after just a single session of choice learning. These findings provide new insights into the learning process of decision-making tasks. They suggest that the value of choice options and the ability to make choices are learned separately and that experience plays a crucial role in improving decision-making performance.

## Significance Statement

We investigated the dynamics of decision-making as rats initially learned to choose between visual stimuli associated with different rewards. Unlike prior research focusing on well-trained participants, we explored the initial stages of learning to make decisions. We used a behavioral design that separated value learning from choice learning. Initially, rats exhibited slower responses when making choices, but this slowing diminished with experience. Response slowing persisted throughout the period of early choice learning. Drift diffusion modeling found reduced evidence of reduced information requirements for making choices over the period of early learning, with decision thresholds decreasing after just one choice-learning session. These studies revealed that experience significantly enhances decision-making and shed light on the learning mechanisms that underlie decision-making tasks.

## Introduction

Neural and computational models of decision-making assume an internal comparative process when participants are faced with multiple options (see Carandini and Churchland, 2013 for review). In studies using two-alternative forced-choice (2AFC) designs, participants are trained to learn the value of simultaneously presented stimuli. Choosing one while forgoing the other may ensure the adoption of a comparative strategy. Few, if any, studies have addressed how decision-making tasks are learned if participants learn the reward values of task stimuli prior to making choices between the stimuli. This is an important issue because the training procedures used for 2AFC tasks could influence the decision-making strategy that is used in later stages of behavioral testing, for example, when neuron recordings are made.

Studies reviewed by Kacelnik et al. (2011) highlight this concern. Their research with European starlings revealed that, in nature, animals typically encounter food options sequentially, not simultaneously as presented in many lab experiments. To better mimic natural foraging in the laboratory, they used a unique design (Shapiro et al., 2008). During training, visual stimuli were presented individually (sequentially) on some trials and together (simultaneously) on other trials. Kacelnik and colleagues found that choices between the stimuli were predicted by the animals’ latencies to respond to the single offers of the stimuli. They further found no differences between the time taken to make a choice between pairs of stimulus and the time taken to respond to offers of just one of the stimuli. Their studies suggest a lack of deliberation, or slowing of response times, on choice trials. This finding was replicated in subsequent studies involving rodents (Ojeda et al., 2018; Ajuwon et al., 2023). Kacelnik et al. (2011) argue that the act of deliberation, observed in lab settings using two-alternative forced-choice (2AFC) designs, might be an artifact of the training process. They further suggest that drift diffusion models (Ratcliff, 1978), which rely on competition between stimulus options, may not be suitable for understanding decision-making. These findings necessitate re-evaluating current interpretations of decision-making research, which often rely on the assumption that deliberation slows down choices and that drift diffusion models adequately capture the underlying neural processes.

Building on the work of Kacelnik et al. (2011), we designed a behavioral task to isolate learning the values of individual stimuli from learning to make choices between stimuli. Using an open-source 2AFC task (Swanson et al., 2021), we first trained rats to learn the reward values of the stimuli. Then, we introduced choice trials with pairs of stimuli and measured how the rats responded when making choices for the first time. Contrary to findings summarized in Kacelnik et al. (2011), responses were initially slower in the first session with choice trials compared with the single-option trials. We continued testing over several sessions to assess the effect of experience on decision-making. Interestingly, the initial slowing during choice trials persisted, suggesting a separate process for decision-making beyond reward learning.

To analyze the decision-making process during initial choice learning, we employed two computational models. First, we used ExGauss models to assess the impact of learning on response time distribution (Hohle, 1965; Luce, 1991). This revealed how learning influenced the overall speed and variability of choices. Second, we employed drift diffusion models to explore if early learning modified parameters within this common neuroscience framework. Our analysis showed that initial learning primarily influenced the decision threshold parameter, not others, within the drift diffusion model. Interestingly, these changes in threshold positively correlated with the exponential variability in response times, which has been linked to noise in the decision process (Hohle, 1965). These findings suggest that early choice learning may lead to needing less information to make a choice and might also reduce noise in the decision process.

## Materials and Methods

### Subjects

Thirty male Long–Evans rats (300–450 g, Charles River, Envigo) were individually housed and kept on a 12 h light dark cycle with lights on at 7:00 A.M. Rats were given several days to acclimate to the facilities, during which they were handled and allowed *ad libitum* access to food and water. During training and testing, animals were on regulated access to food to maintain their body weights at ∼90% of their *ad libitum* access weights. All animal procedures were approved by the American University Institutional Animal Care and Use Committee.

Only male rats were used in this study. The behavioral data were collected prior to the implementation of the NIH policy that studies use equal numbers of male and female animals in research projects. The experiments were supported by non-NIH sources of funding. Support from an NIH grant was later in the project when the data analysis was carried out and the manuscript was written.

### Behavioral apparatus

All animals were trained in sound-attenuating behavioral boxes (ENV-018MD-EMS: Med Associates). A single horizontally placed spout (5/16″ sipper tube: Ancare) was mounted to a lickometer (Med Associates) on one wall, 6.5 cm from the floor and a single white LED light was placed 4 cm above the spout (henceforth referred to as the spout light). The opposite wall had three 3D-printed nosepoke ports aligned horizontally 5 cm from the floor and 4 cm apart, with the IR beam break sensors on the external side of the wall (Adafruit).

Three Pure Green 1.2″ 8 × 8 LED matrices (Adafruit) were used for visual stimulus presentation and were placed 2.5 cm above the center of each nosepoke port, outside the box (see Swanson, et al., 2021 for details about these matrices). Data collection and behavioral devices, including the Arduino that interfaced with the LED matrices, were controlled using custom-written code for the MedPC system, version IV (Med Associates).

### Training procedure

**Figure 1.**
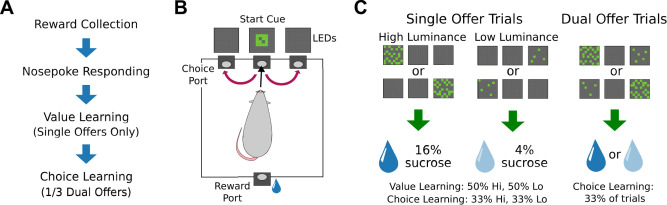
Task training, design, and trial types. ***A***, Rats went through a series of intermediate training steps to learn the task protocol, including how to collect the liquid sucrose reward and nosepoke to gain access to said reward, and learn cue values (“value learning”) for several sessions before experiencing “choice learning”. ***B***, Animals responded to visual cues on one side of the operant chamber and crossed the chamber to consume liquid sucrose at the reward spout. We measured response latency as the time elapsed between trial initiation (center poke) to nosepoking the port with the visual stimulus (red arrows). ***C***, For the value learning phase, rats only had access to single-offer trials of either high or low luminance stimuli. Upon entering the central port on these trials, one cue would appear above either the left or right nosepoke ports, randomized by location and value. Responding to the high luminance stimulus led to access to 16% liquid sucrose at the reward port. Responding to the low luminance stimulus led to access to 4% liquid sucrose. During testing in the choice learning phase, 2/3 of the trials consisted of these single-offer trials. For the remaining 1/3 of trials, rats were exposed to dual-offer trials with both high and low luminance cues displayed. Single and dual offers were randomly interleaved.

Animals were initially exposed to 16% sucrose in their homecage to encourage consumption and reduce novelty to the reward during operant training. Rats were then introduced to the operant chambers and trained to lick at a reward spout for 16% wt/vol liquid sucrose in the presence of a spout light and 0.2 s 4.5 kHz SonAlert tone (Mallory SC628HPR). One rat was dropped at this point in the training for lack of interest in consuming sucrose. Over the next several sessions, animals experienced training for reward collection (Fig. 1*A*). They were hand-shaped to respond to visual stimuli over lateralized nosepoke ports to gain access to a 50 µl bolus of liquid sucrose reward at the spout on the opposite side of the chamber. A correct nosepoke (responding at the illuminated port) was indicated by the tone and spout light illumination. These trials comprise the “nosepoke responding” phase of training (Fig. 1*A*). Two rats were dropped from the protocol at this stage of training for not completing >120 trials in a 60 min session within five sessions. See Table 1 for a summary of the training procedure and criterion to advance.

**Table 1.** Training paradigm and criterion to advance rats to the next stage of testing

Stage	Name	Goal	Stimulus + reward	Criterion
1	Continuous Access	Pair spout light and tone with reward	No stimulus; 50 µl 16% liquid sucrose bolus	>500 licks per session
2	Nosepoke Training—left or right side only	Rats nosepoke in operant ports following high luminance stimulus	8 LEDs over the left or right operant port. Trials only appear on one side port with alternative port blocked. Correct response triggers spout light and tone, indicating availability of 16% sucrose reward	>120 trials/session; 3 session minimum, 6 session maximum
3	Random cue presentation	Rats respond to the visual cue presentations	8 LEDs presented at random over the L/R operant port. Correct response triggers spout light and tone indicating availability of 16% sucrose reward. Error response to the nonilluminated operant port triggers the spout light requiring response at reward port, but no reward delivered	>200 trials/session; <10% error trials; 3 sessions minimum, 6 sessions maximum
4	Center cue training	Encourage rats to initiate trials by responding to the center “start” cue	Start stimulus presented at the center, followed by random presentation of high luminance stimulus over the L/R operant port. Correct and error responses remain the same	>200 trials/session; <10% error trials; 3 sessions minimum, 6 sessions maximum
5	Single-offer only sessions	Rats are introduced to the low luminance cue and associate it with low-value reward	Start cue presented at the center, followed by random presentation of either the high luminance or low luminance cue over the L/R operant port. Response triggers spout light and tone, indicating availability of 16% or 4% sucrose reward, paired to the luminance	10 sessions (±2); established response time difference for the high and low response latencies
6	Single- and dual-offer sessions	Final stage of the task	Start cue presented at the center, followed by random presentation of either the high luminance or low luminance cue over the L/R operant port for 2/3 trials. For 1/3 trials, both H/L stimuli are presented over L/R operant ports	1 session to 5 sessions of testing

Rats that reached these criteria were advanced to the next stage of training where a trial initiation cue (4 × 4 square of illuminated LEDs) was introduced over the central nosepoke. After rats entered the central port, a single stimulus of either high (eight illuminated LEDs) or low luminance (two illuminated LEDs) was presented without delay. These trials comprise the “value learning” phase of training (Fig. 1*A*). These stimulus presentations were randomized by side and luminance intensity and importantly were only offered one stimulus at a time (single-offer trial). Correct responses at the port below the high luminance stimulus yielded access to a 16% wt/vol sucrose bolus at the reward spout, while responses at the port below the low luminance cue response yielded access to a 4% wt/vol sucrose bolus. The tone and light were once again indicative of access to these sucrose rewards. Incorrect responses at this stage in the task were indicated only by the presence of the spout light (no tone), and rats were required to make contact with the reward spout before initiating the next trial. Subjects were self-paced throughout the period of training and testing.

When subjects performed >200 trials per 60 min session during this training phase with fewer than 10% errors, they were moved to the testing phase, “choice learning,” where they were introduced to dual-offer trials (Fig. 1*A*). Two rats were dropped at this stage of training for aberrant strategies (circling all three nosepoke ports), so a total of 25 rats moved on to the testing phase. Ten of these rats were tested in a single session with choice learning. The rest of the rats (*N* = 15) were tested over five choice sessions, with sessions with only single-offer stimuli interleaved over days.

Two types of trials were included in the choice learning sessions. On single-offer trials, animals were presented with a single stimulus randomized by side and luminance intensity. Single-offer trials comprised 2/3 of total trials per testing session. On dual-offer trials, which comprised 1/3 of total trials, animals were presented with both the high and low luminance stimuli. The presentation of the brighter stimulus was randomized by side to prevent a spatial strategy in responding on these dual-offer trials.

Response latency was measured as the time elapsed between rats entering the central port to their entrance to their chosen side port after the onset of the visual stimuli. On single-offer trials, we defined errors as entering the nonilluminated port. On dual-offer trials, we determined the choice percentage to assess the preference subjects had for high- and low-value sucrose.

### Data analysis: software and statistics

Behavioral data were saved in standard MedPC data files (Med Associates) and were analyzed using custom-written code in Python and R. Analyses were run as Jupyter notebooks under the Anaconda distribution.

Statistical testing was performed with R and the scipy, pingouin, and DABEST packages for Python. Repeated-measures ANOVA (with the error term due to subject) were used to compare behavioral data measure estimates (median latency, high choice percentage, ExGauss parameters, and DDM parameters from the PyDDM package—see below) across trial type (single or dual offer), value (high or low), and/or session number (for rats tested over five sessions). For significant rmANOVAs, the error term was removed and Tukey’s post hoc tests were performed on significant interaction terms for multiple comparisons. Descriptive statistics are reported as mean ± SEM, unless noted otherwise. Two-sided, paired permutation tests were used to compare single and dual offers within each session (Ho et al., 2019) with Bonferroni corrected *p* values. A total of 5,000 bootstrap samples were taken; the confidence interval was bias corrected and accelerated. The *p* values reported are the likelihood of observing the effect size if the null hypothesis of zero difference is true. For each *p* value, 5,000 reshuffles of the control and test labels were performed. Results are displayed as summaries with individual points produced by Matplotlib and Seaborn. Within session effects of trial time and response latency were analyzed with Huber regression from the scikit-learn package for Python. Relations among the parameters from ExGauss modeling and PyDDM were examined using the regplot function from the Seaborn package. Results were quantified using pairwise correlation (Spearman’s rank) using the pairwise_corr function from the pingouin package. Repeated-measures correlation, for the effect of training sessions, was quantified using the rm_corr function from the pingouin package.

### Data analysis: behavioral measures and computational models

Response latency was defined as the time elapsed from the initiation of a trial to the nosepoke response in the left or right port. Response latencies >3 s were screened out of the data to exclude trials where rats were disengaged from the task. Median response latency was calculated per rat. Choice percentage reflects the rate at which rats responded to the high luminance target on dual-offer trials. This was calculated by dividing the number of high luminance trials by the total number of dual-offer trials per rat. Errors on single-offer trials occurred when rats produced a nosepoke response in a nonilluminated port. Error percentages were calculated by dividing the number of error trials by the total number of single-offer trials per rat.

#### ExGauss modeling of response latencies

**Figure 2.**
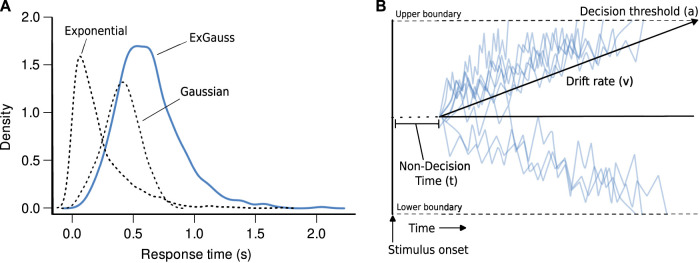
ExGauss and drift diffusion modeling. ***A***, ExGauss models estimate response time distributions as mixtures of Gaussian and exponential distributions. The ExGauss distribution is the sum of the Gaussian and exponential components. In the example shown here, parameters from one of the rats were used to simulate full distributions for each component. The Gaussian component accounts for the peak in the response time distribution. The exponential component accounts for the long positive tail in the distribution. ***B***, Drift diffusion models, or DDMs, estimate three parameters of the decision process based on random fluctuations of evidence for the response options over the time from the onset of the stimuli to the time of choice. The drift rate reflects the slope of the accumulation of evidence toward the threshold, when a choice is triggered. Nondecision time accounts for stimulus integration and sensorimotor processing.

Due to the right-skewed nature of response latencies, we wanted to assess how latency distributions change over repeated sessions. Here we used ExGauss model fitting to analyze the distributions. ExGauss is a mixture model of a Gaussian (normal) distribution and exponential distribution, the latter of which captures the extended tail of the response latencies (Fig. 2*A*). The retimes library for R was used for the ExGauss analysis. This package is based on Cousineau et al. (2004). The timefit() function was used to fit an ExGauss model to data for individual rats for each session number (five sessions); number of LEDs (two or eight); and trial type (single and dual offers). To assess the accuracy of fit of these models to the raw data, especially in cases of a smaller dataset for low-value dual-offer trials, we used the ExGauss() function to generate data from the parameter fits and compared the generated and raw data with a Kolmogorov–Smirnov test was used. Any significant differences (*p* < 0.05) between raw and generated data distributions were further tested with a nonparametric ranksums test. No significant differences were found between the datasets, suggesting the ExGauss parameters were fair to represent the raw distributions. To further validate this toolbox, we also used the exgfit toolbox in MATLAB and found no difference in the parameter fits.

#### Drift diffusion model fitting using the HDDM package

The HDDM package (Wiecki et al., 2013; version 0.9.6) was used to quantify effects of learning on the three mean DDM parameters, drift rate, decision threshold, and nondecision time (Fig. 2*B*). Drift rate accounts for how quickly the rats integrate information about the stimuli. Threshold accounts for how much information is needed to trigger a decision. Nondecision time accounts for the time taken to initiate stimulus integration and execute the motor response (choice). A fourth parameter that can be included in HDDM models is called bias. It accounts for variability in the starting point of evidence accumulation. We used a fixed bias of 0.5 for the main analyses reported in this study.

HDDM models were fit that allowed for a single DDM parameter (drift rate, threshold, nondecision time) to vary freely over sessions. The other parameters were estimated globally using data from dual-offer trials in all sessions. Parameters were from Pedersen et al. (2021): models were run five times, each with 5,000 samples and the first 2,500 samples were discarded as burn-in. Convergence was validated based on the Gelman–Rubin statistic (Gelman and Rubin, 1992). The autocorrelations and distributions of the parameters for each parameter and predictions of the response latency distributions for each animal were visually assessed to further assess convergence. HDDM is sensitive to outliers (Wiecki et al., 2013), so we included latencies up to the 95th percentile of the distribution in our analyses. Exploratory data analysis found that the 95th percentile cutoff was approximately the same for the response time distributions across learning sessions.

#### Drift diffusion model fitting using the PyDDM package

The PyDDM package in Python (Shinn et al., 2020) was used to fit generalized drift diffusion models to these data. PyDDM does not use hierarchical Bayesian modeling. It is an algorithmic approach, based on the Fokker–Planck equation (Shinn et al., 2020). An advantage of using PyDDM was that we could obtain estimates of the decision parameters for each rat and compare them with other behavioral measures.

We fit an initial model to the response latency distribution from all rats for each session under the method of differential evolution. For our model, we fit a total of three parameters: drift rate which was linearly dependent upon the log of the measured luminance of the high and low stimuli [drift*log(luminance)], boundary separation, and nondecision time. Our model also accounted for three constant parameters: noise, or the standard deviation of the diffusion process, set to 1.5; initial condition set to 0.1 to account for initial bias toward the high-value stimulus; and a general baseline Poisson process lapse rate set to 0.2 specified by the PyDDM package for likelihood fitting. Once this model was tuned to the group data (to identify which parameters may be dependent on task parameters), we fit the model to data from individual rat data from each session to be able to analyze individual shifts in parameters. A repeated-measures ANOVA was performed to assess learning effects on the fitted parameters (drift, boundary separation, and nondecision time) and post hoc tests used permutation methods from the estimation statistics package DABEST (Ho et al., 2019).

### Data sharing

Data files are available on GitHub: https://github.com/LaubachLab/LearningToChoose.

## Results

### Rats deliberate when making choices for the first time

**Figure 3.**
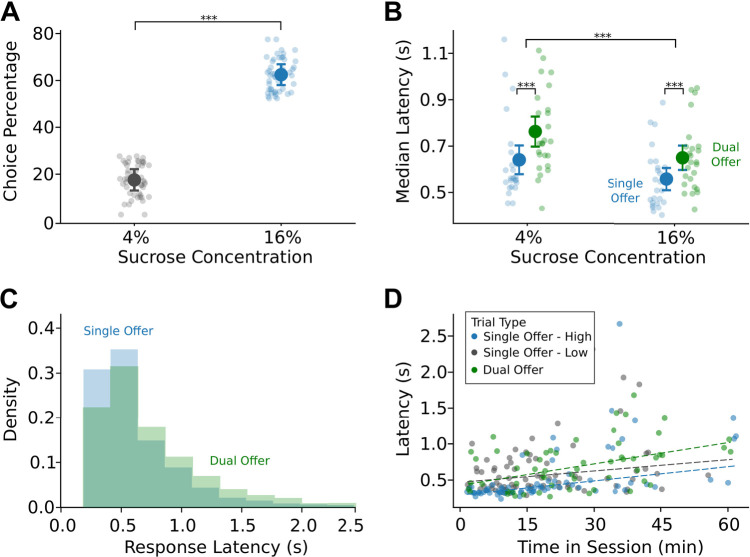
Rats deliberate when making choices for the first time. ***A***, On dual-offer trials, rats (*N* = 25) selected the high luminance, high-value cue ∼72% of the time overall, suggesting these subjects prefer the high-value reward over the low-value option. ***B***, Rats showed overall increased latencies for low-value offers compared with high-value offers. Importantly, rats showed increased latencies for dual-offer trials compared with single-offer trials regardless of the chosen value. ***C***, Raw latency distributions for trials with single and dual offers showed a nonoverlapping portion in the tail ends of the distribution. ***D***, Response latencies generally increased over the session across trial types and values (data from one exemplar rat shown).

We sought to investigate whether training rats with single-offer trials and then exposing them to dual-offer trials with known stimuli would produce behavioral changes associated with deliberation. After training for multiple sessions with single-offer trials, rats show marked differences in behavior on dual-offer trials. In a 1 h session, rats on average completed 301 ± 74 trials. Rats chose the high-value stimuli (72% ± 6%) more often than the low-value stimuli (28% ± 6%) when both options were presented during the initial test session (*t*_(24)_ = −16.16; *p* < 0.001; paired *t* test; Fig. 3*A*). We also found that rats show an increased median response latency for trials with dual offers compared (625 ms) with trials with single offers (521 ms) (*F*_(1,24)_ = 37.36; *p* < 0.001; rmANOVA) and for trials with low-value stimuli (597 ms) compared with trials with high-value stimuli (527 ms; *F*_(1,24)_ = 28.31; *p* < 0.001; rmANOVA; Fig. 3*B*). Further, when broken out by value, the difference between dual- and single-offer median latencies was significant for high-value trials (mean difference: 0.09; *p* < 0.001; paired permutation test) and low-value trials (mean difference: 0.12; *p* < 0.001; paired permutation test). When looking at the response distributions, we found that trials with single and dual offers generally had nonoverlapping exponential tail distributions (Fig. 3*C*). Finally, we assessed within-session effects on latencies and found there was a general effect of slowing over the one hour session (1H: weighted *R*^2 ^= 0.25; *F*_(1,78)_ = 4.397; *p* < 0.001; 1L weighted *R*^2 ^= 0.42; *F*_(1,72)_ = 4.082; *p* < 0.001; 2H: weighted *R*^2 ^= 0.17; *F*_(1,57)_ = 4.563; *p* < 0.001; robust M-regression; Fig. 3*D*).

### Deliberation is persistent over the period of early choice learning

**Figure 4.**
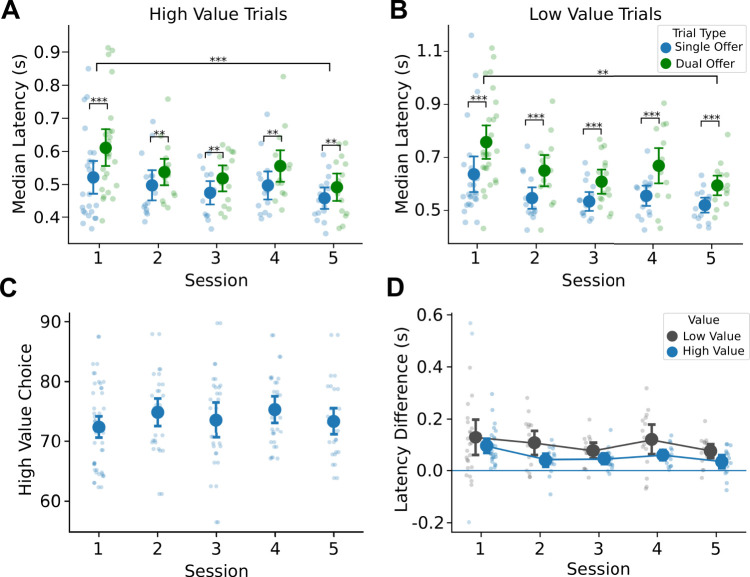
Rats reduce their response latencies but maintain deliberation with experience in making choices. ***A***, Median response latencies for high-value trials from 15 rats showed reduction with experience while maintaining an increase on dual-offer compared with single-offer trials. ***B***, Median response latencies for low-value trials were overall greater than high-value trials and showed reduction with experience while maintaining an increase on dual-offer compared with single-offer trials. ***C***, Rats did not change their proportion of high-value choices on dual-offer trials with more experience. ***D***, The magnitude of difference in single- and dual-offer median latencies reduced with experience, especially from the first to second session; however, subjects maintained an elevated latency for dual-offer trials over the course of choice learning.

Given the robust effect that we found with respect to latency differences from trials with single and dual offers, we wanted to examine how stable the deliberation effect was with repeated testing. Fifteen rats were tested over five sessions of choice learning (Fig. 4). Given the overall differences in latency between high- and low-value stimuli (*F*_(1,14)_ = 87.06; *p* < 0.001), we broke out trials by value and used Bonferroni’s corrections for tests of significance. In the case of high-value trials, we found an overall effect of session number on median latencies (*F*_(1,14)_ = 6.953; *p* < 0.001; rmANOVA) and an overall effect of trial type (*F*_(1,14)_ = 34.607; *p* < 0.001; rmANOVA).

To further investigate the differences in latencies for trials with single and dual offers, we used paired permutation tests with Bonferroni’s correction given this test is robust for relatively small sample sizes. We found that the difference in median latencies from trials with single and dual offers persisted over the five sessions (Fig. 4*A*; paired permutation tests). For low-value trials, we found similar results; overall effect of session number on median latencies (*F*_(1,14)_ = 4.593; *p* = 0.00168; rmANOVA), an overall effect of trial type (*F*_(1,14)_ = 57.750; *p* < 0.001; rmANOVA), and the difference in median latencies from trials with single and dual offers persisted over the five sessions with choice learning (Fig. 4*B*; paired permutation tests). These changes in median response latency occurred in the absence of an effect on high-value choice percentage (*F*_(1,14)_ = 2.339; *p* = 0.0585; rmANOVA; Fig. 4*C*).

To emphasize the effect that dual-offer trials have on response latencies, we analyzed the median difference in trial types by value. We confirmed that these latency differences persisted over the five sessions with choice learning. However, there was a greater latency difference in the first session compared with the subsequent sessions (high value: *F*_(1,14)_ = 3.171; *p* = 0.0203; low value: *F*_(1,14)_ = 3.503; *p* = 0.0127; rmANOVA; Fig. 4*D*).

### ExGauss modeling of response time distributions

Given the skewed nature of the latency distributions (Fig. 3*C*), we used ExGauss modeling (Heathcote et al., 1991) to separate the latency distributions into a Gaussian component (mu parameter) that account for the peaks in the distributions and an exponential component (tau parameter) that account for the positively skewed tails in the distributions (Fig. 2*A*). These measures have been interpreted, respectively, as reflecting sensorimotor processing and variability in the decision process (Hohle, 1965; Luce, 1991).

**Figure 5.**
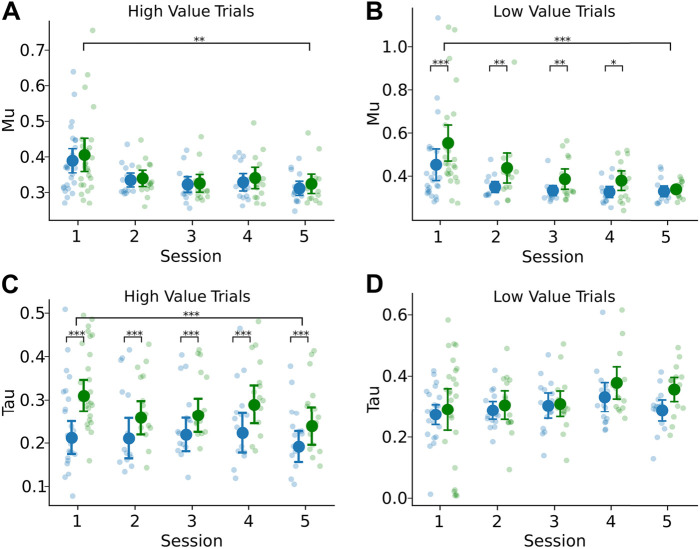
ExGauss modeling reveals differences in response latency distributions for dual- and single-offer trials. ***A***, The ExGauss parameter Mu represents the mean of the Gaussian component, a measure of sensorimotor integration. An increase in this parameter indicates an overall shift in the distribution to the right. There was no difference for single and dual trial fits of the Mu parameter for high-value trials; however, there was an overall reduction in the Mu parameter over all the sessions. ***B***, In the case of low-value trials, there was an overall increase of the Mu parameter on dual-offer trials and a decrease in this parameter over all the sessions. ***C***, The ExGauss parameter Tau represents the mean of the exponential component, a measure of variability in the decision process. An increase in this parameter indicates an overall lengthening of the distribution tail to the right. There was an overall increase of the Tau parameter on dual-offer trials for the high-value stimulus and a decrease in this parameter over all the sessions. ***D***, In the case of low-value trials, there was no difference for single and dual trial fits of the Tau parameter and no change over the sessions.

We fit ExGauss models to the response latency distribution of each rat for each learning session and each type of trial. We found that the rats showed more variable latencies when choosing the high-value stimulus compared with when they were forced to respond to the stimulus (Fig. 5*A*,*C*). In contrast, they responded more slowly, with equal variability, when choosing the low-value stimulus compared with forced responses to that stimulus (Fig. 5*B*,*D*). For high-value trials, we found an overall effect of session number on the Gaussian component (*F*_(1,14)_ = 4.170; *p* = 0.00328; rmANOVA; Fig. 5*A*), but no overall effect of trial type (*F*_(1,14) _= 2.915; *p* = 0.09016; rmANOVA). In the case of low-value trials, however, we found an overall effect of both sessions (*F*_(1,14)_ = 5.076; *p* < 0.001; rmANOVA) and trial type (*F*_(1,14)_ = 18.438; *p* < 0.001; rmANOVA; Fig. 5*B*). Paired permutation tests further reveal a persistent difference between trials with single and dual offers over the first four sessions.

When examining the response latency variability in the exponential tail, we found the opposite effect with respect to value. For high-value trials, we found an overall effect of session (*F*_(1,14)_ = 4.254; *p* = 0.00287; rmANOVA) and an overall effect of trial type (*F*_(1,14)_ = 37.559; *p* < 0.001; rmANOVA; Fig. 5*C*). Paired permutation tests further reveal a persistent difference between trials with single and dual offers over the five sessions. In the case of low-value trials, there was no effect of session (*F*_(1,14)_ = 1.651; *p* = 0.165; rmANOVA) or trial type (*F*_(1,14) _= 2.553; *p* = 0.113; rmANOVA) on the exponential variability (Fig. 5*D*).

### Drift diffusion modeling of decision dynamics

**Figure 6.**
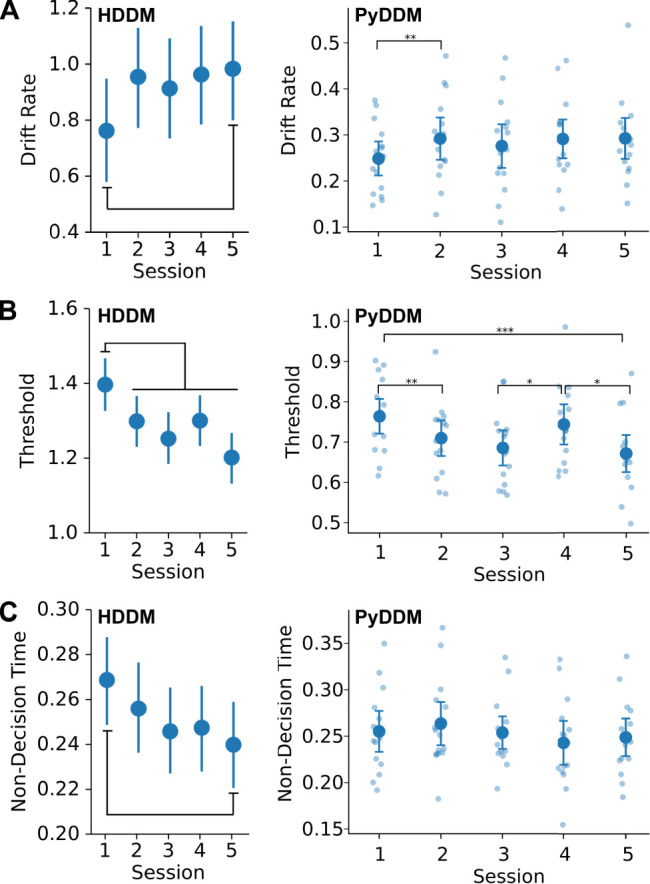
Rats require less evidence to make choices during early learning. ***A***, Drift rate increased after the first session of choice learning. ***B***, Threshold reduced over the period of choice learning and importantly was reduced after a single test session. ***C***, Nondecision time based on HDDM was higher in the first test session compared with the fifth session. No effects of learning on nondecision time were found using PyDDM. Please note that the two kinds of DDMs report the decision parameters using different units.

To measure how early choice learning affected cognitive processes that underlie decision-making, we used two kinds of drift diffusion models, hierarchical Bayesian drift diffusion modeling (HDDM; Wiecki et al., 2013) and generalized drift diffusion modeling (PyDDM; Shinn et al., 2020). A common finding across models was that initial choice learning reduced the decision threshold (Fig. 6), even after a single session of choice learning. The left plots in each panel in Figure 6 show Bayesian estimates of the mean and 95% confidence intervals for the DDM parameters from analysis using HDDM. Differences are noted based on comparisons of the posterior distributions for each parameter, comparing the first session against the rest. Drift rate was lower in the first learning session compared with the fifth session [*p*(1 > 5): 0.0456; Fig. 6*A*, left]. Threshold was higher in the first learning session compared with all other sessions [e.g., *p*(1 < 2): 0.0204; Fig. 6*B*, left]. Nondecision time was longer in the first learning session compared with the fifth session [*p*(1 < 5): 0.0172; Fig. 6*C*, left].

Figure 6 also shows results from the PyDDM models. We found no overall effect of session number on the drift (Fig. 5*B*; *F*_(1,14)_ = 1.979; *p* = 0.11; rmANOVA) or nondecision time (Fig. 5*D*; *F*_(1,14) _= 1.382; *p* = 0.252; rmANOVA) parameters. We did, however, find a significant increase in drift from the first to second session before the drift stabilizes for the remaining sessions (paired mean difference: 0.044; *p* = 0.0078). We found an overall effect of the boundary separation parameter (Fig. 5*C*; *F*_(1,14)_ = 4.316; *p* = 0.0041; rmANOVA), with differences from session to session (Fig. 5*C*).

### Relationships between measures of choice behavior and the drift diffusion models

**Figure 7.**
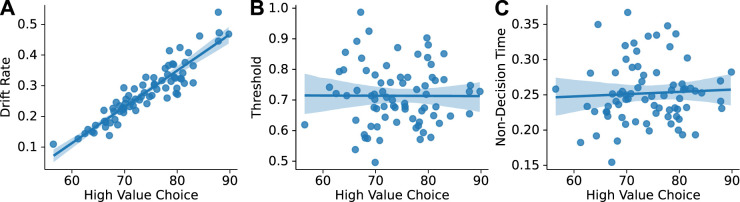
Drift rate is driven by choice preference. Scatterplots are shown for the high-value choice percentage across animals and sessions versus the three parameters from the PyDDM models. Regression lines were fit with 95% confidence intervals. Correlational values were calculated using repeated-measures correlation to control for effects of training sessions. ***A***, There was a strong positive relationship between choice preference and drift rate. ***B***, ***C***, There was no clear relationship between choice preference and threshold or nondecision time.

We used repeated-measures correlation to assess how the behavioral and computational measures reported above related to each other. For this analysis, we used the parameters from the PyDDM models and related them to the animals’ preferences for the higher value stimulus and measures of their response times based on ExGauss modeling. The strongest correlation across measures was between the drift rate and the percent of trials in which the rats chose the higher value stimulus (*r* = 0.9222; df = 69; *p* < 0.001; CI95%: 0.88–0.95; Fig. 7*A*).

Threshold and nondecision time did not covary over animals in relation to the animals’ choice preferences (Fig. 7*B*,*C*). This finding suggests that drift rate was driven by the animals’ preferences for the 16% liquid sucrose reward and that the amount of information needed to make a choice or the time taken for sensorimotor processing was not related to the animal’s preferences.

Two other behavioral measures were also evaluated. One was the difference in median latency for trials with low- and high-value stimuli. This difference is a proxy for the effect of reward value on the rats’ response latencies. The other was the difference between median latencies for responses to the high-value stimulus on dual and single-offer trials. This difference is a proxy for the effect of choice on the response latencies. These measures did not have large pairwise correlations with any of the DDM parameters. The only significant correlations were between the proxy for choice and the threshold parameter (*r* = 0.5237; df = 69; *p* < 0.001; CI95%: 0.33–0.67) and the proxy for value and the drift rate parameter (*r* = 0.3715; df = 69; *p* < 0.002; CI95%: 0.15–0.56). These positive relationships suggests that threshold was higher in rats that were slower to respond to the high-value stimuli on dual-offer trials and drift rate was higher in rats that were slower to respond on dual-offer trials compared with single-offer trials.

### Relationships between parameters from the ExGauss and drift diffusion models

**Figure 8.**
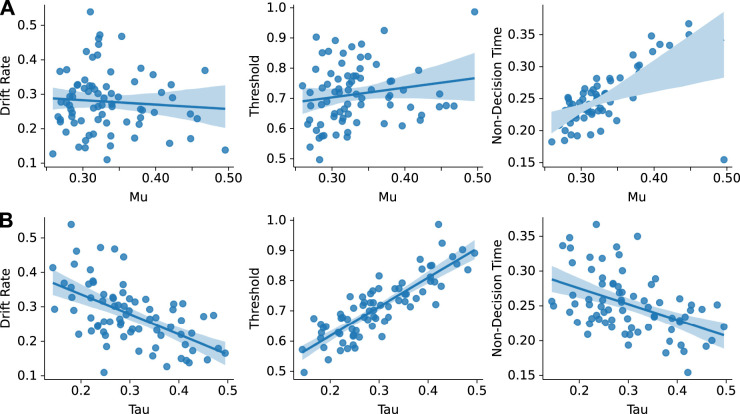
ExGauss parameters had distinct relations to the DDM parameters. ***A***, The Mu parameter from ExGauss modeling was showed a positive relationship nondecision time but not the other DDM parameters. ***B***, The Tau parameter from ExGauss modeling showed strong relationships to all three DDM parameters across animals and sessions.

Another strong pairwise correlation was found between the Mu parameter (Gaussian) from the ExGauss models and the nondecision time parameter from the DDMs (*r* = 0.6890; df = 69; *p* < 0.01; CI95%: 0.54–0.79; Fig. 8*A*). Interestingly, the other two parameters from the DDMs did not covary with the Mu parameter. Even more dramatic correlations were observed between the Tau parameter (exponential) from the ExGauss models and all three parameters from the DDMs (Fig. 8*B*). Threshold was strongly positively related to Tau (0.8330; df = 69; *p* < 0.001; CI95%: 0.74–0.89), and therefore threshold was highest in animals that showed the largest exponential variability in their response times. Drift rate and nondecision time showed somewhat weaker negative relations to Tau (drift rate: −0.5606; df = 69; *p* < 0.001; CI95%: −0.70 to −0.38; NDT: −0.4848; df = 69; *p* < 0.001; −0.65 to −0.28), meaning that drift rates and nondecision times were lowest in animals with high levels of exponential variability.

## Discussion

We investigated the initial acquisition of a decision-making task using a unique training protocol. We trained the reward values of task stimuli by presenting the stimuli as single offers. Then, we tested animals with dual offers on one-third of the trials and measured how rats initially learned to make choices. Initially, rats took longer to decide on dual-offer trials but improved their speed of choice over time. However, they still took longer to make choices compared with single-offer trials throughout the period of testing. To understand the cognitive mechanisms of early choice learning, we fit drift diffusion models and found that initial choice learning reduced the amount of information needed to trigger a choice. We suggest that brain systems related to the control of the decision threshold may undergo learning-related changes during early learning. One potential candidate brain region is the anterior cingulate cortex (Domenech and Dreher, 2010).

### First instance of choice impacts behavior

During the first session with dual-offer trials, rats showed a preference for high-value cues (Fig. 2*A*). Additionally, they showed increased response latencies for dual-offer compared with single-offer trials for both high- and low-value trials (Fig. 2*B*). These results suggests rats deliberate (i.e., show slowing on dual-offer trials compared with single-offer trials) and likely engage in some comparative process when deciding between options of known value. Our findings are quite different from the studies by the Kacelnik group that motivated our experiment [Shapiro et al. (2008), their Fig. 6; Ojeda et al. (2018), their Fig. 2; Ajuwon et al. (2023), their Fig. 4]. Their studies reported much longer overall response latencies (on the order of seconds). It is possible that the longer latencies reflected a lack of speeded performance and therefore their studies would not reveal an effect of deliberation that is on the order of milliseconds. Our rats responded with median latencies between 520 and 625 ms and showed slowing of ∼100 ms on dual-offer trials with responses to high-value stimuli (Fig. 2). An increase in response latency of 100 ms on dual-offer trials added 20% more time to make choices, an effect that is not trivial.

Given the magnitude of difference for latencies to single and dual offers in the first test session, we assessed the stability of that difference and if/how decision-making might change with repeated experience. We found there was no significant change in rate of high-value choices on dual-offer trials over five sessions with choice learning (Fig. 3*C*), suggesting the rats were not relearning the value of each cue when they were presented simultaneously. Their preferences were consistent with the Matching Law (Herrnstein, 1961): the fourfold difference in sucrose concentration used in our study predicts a 75% high-value preference (Graft et al., 1977). Over five sessions with dual-offer trials, median response latencies reduced, suggesting that the rats learned to choose more quickly (Fig. 3*A*,*B*). To determine if response slowing was sensitive to experience, we calculated the difference between single-offer and dual-offer median response latencies. We found that the difference was most robust in the first test session. While the magnitude of the difference decreased with learning, the effect of deliberation persisted over the five sessions for both reward values (Fig. 3*D*).

Previous studies of visual decision-making in rodents have not commonly used the two-stage design as in the present study. Some of these studies trained animals to detect single stimuli and to report the identity of the stimulus by responding to the left or right, i.e., object–place learning (Zoccolan et al., 2009; Kurylo et al., 2020; Masis et al., 2023). Others trained rodents to make lateralized movements toward the chosen stimulus and trained discrimination between stimuli from the start of initial training (Clark et al., 2011; Reinagel, 2013; Broschard et al., 2019; Kurylo et al., 2020; Broschard et al., 2021; Liebana Garcia et al., 2023; Masis et al., 2023). The only published study that we found that trained rodents with single stimulus presentations before choice learning was Busse et al. (2011). However, that study did not report results on how early task learning affected response latencies.

The changes in deliberation over early choice learning are interesting in the context of many neuroscience studies of decision-making that used extensively overtrained animals (see Carandini and Churchland, 2013 for review). If a given brain area is only involved in the acquisition of choice learning, it is possible that it would not show major changes in neural activity once the animals become overtrained. As an example, Katz et al. (2016) reported a lack of decision-related firing in area MT in monkeys performing a motion discrimination task. However, if this cortical area was inactivated, the monkeys showed robust impairments in task performance. Area MT is well established as containing neurons that track visual motion. It is possible that neurons in that cortical area were dramatically altered during the acquisition of the motion discrimination task and become less engaged after extensive overtraining.

### Choice learning reduced exponential variability

Given the distributions of response latencies deviated from the Gaussian distribution (Fig. 2*C*), we implemented a mixture model to estimate the peaks and long tails of the response latency distributions (ExGauss modeling; Heathcote et al., 1991). For high-value trials, there was no difference in the Mu parameter, representing the peak in the response latency distribution (Fig. 5*A*). In contrast, low-value trials showed increased peak latencies for dual-offer trials compared with single-offer trials, over the first four sessions with choice learning (Fig. 5*B*). This finding is suggestive of an overall slowing of when rats chose the low-value stimulus.

The other main ExGauss parameter Tau represents the variability in the tail of the distribution, which might be due to decision variability, aka “noise” in the decision process (Hohle, 1965). Tau was consistently elevated on high-value dual-offer trials compared with high-value single-offer trials throughout the period of choice learning (Fig. 5*C*). Exponential variability decreased over the course of the five choice learning sessions, but the difference between trial types remained significant throughout, suggesting decision variability is elevated on high-value choice trials regardless of the stage of learning. However, in the case of the low-value stimulus, there was no difference for single and dual trial fits of the Tau parameter and no change over the sessions (Fig. 5*D*), so decision variability seemed to only affect high-value choices. Taken together, the two main parameters of the ExGauss models dissociated the higher- and lower-value trials, a finding that suggests a fundamental difference in how stimuli with different reward values are processed by rats.

### Choice learning reduced the decision threshold

To gain insights about how decision-making strategies might change over the course of sessions with dual-offer trials, we fit drift diffusion models to our data (Fig. 6). We used two established packages for fitting DDMs, HDDM (Wiecki et al., 2013), and PyDDM (Shinn et al., 2020). We found that the threshold, or boundary separation, was the only parameter that changed over the course of the five sessions in both types of DDM models. This parameter reflects the amount of evidence required to make a choice (Ratcliff, 2001). Our findings suggest that the rats came to require less evidence to respond with repeated experience in making choices. The finding that drift rate did not change is not surprising given that preference for the high-value stimulus was stable throughout early choice learning and that there was a strong correlation between drift rate and stimulus preference (Fig. 7*A*) and not the other parameters of the DDM models (Fig. 7*B*,*C*).

Threshold has been associated with caution in performing challenging tasks under time pressure (Forstmann et al., 2008), a process that would depend on inhibitory control. Several recent studies have implicated inhibitory processing in decision-making, specifically with regard to the maintenance of the decision threshold (MacDonald et al., 2017; Roach et al., 2023). A reduction in inhibitory control would lead to a generalized speeding of performance. While there was an overall decrease in median response latency over the period of training (Figs. 2, 3), the results from ExGauss modeling (Fig. 5) do not support a role of inhibitory control in choice learning. Specifically, for trials with high-value stimuli, we observed increased exponential, but not Gaussian, variability compared with single-offer trials. In contrast, we observed increased Gaussian, but not exponential, variability for trials with choices of low-value stimuli. It is not easy to understand how a common process such as inhibitory control would lead to this dissociation in the Gaussian and exponential components of the latency distributions.

A simpler explanation supported by our findings is that early choice learning reduced noise in the decision process. Reductions in threshold were coupled with reductions in exponential variability when rats chose the higher value stimulus. These changes together suggest that rats came to act more quickly when making choices due to their need for less information about which stimulus to choose processed by a more reliable decision-making system.

## Synthesis

Reviewing Editor: Jibran Khokhar, Western University Department of Anatomy and Cell Biology Decisions are customarily a result of the Reviewing Editor and the peer reviewers coming together and discussing their recommendations until a consensus is reached. When revisions are invited, a fact-based synthesis statement explaining their decision and outlining what is needed to prepare a revision will be listed below. The following reviewer(s) agreed to reveal their identity: Kurt Fraser. Note: If this manuscript was transferred from JNeurosci and a decision was made to accept the manuscript without peer review, a brief statement to this effect will instead be what is listed below.

Both reviewers felt that this was an elegant study and have minor comments which should be easily addressable. Additional focus on the choice of approach for the modelling as some additional interpretation of the findings and the potential limitations would be great. I have appended the comments in full from the reviewers to both give you a sense of the congratulatory nature of the remarks but also the specific comments to address.

Reviewer 1

This is an elegant study from White et al., that investigates the computational mechanisms underlying the acquisition of 2 alternative forced choice-style task. The study is motivated by a paucity of prior research on the acquisition and refinement of decision-making mechanisms versus the rich literature on what computational bases underlie choice after learning. The authors trained male rats to associate visual stimuli with the delivery of either 4% or 16% sucrose and then pitted these options as choices. The authors demonstrate that in these choice trials the rats spend longer to execute their choice and that the speed of deciding increases across training, that is the rats deliberate less as they become better decision-makers. They then model this behavior with either an exponential gaussian approach or with drift diffusion models. One of the most exciting findings is that preference for the high value option was correlated with drift rate which suggests a direct relationship between individual differences in valuation with decision making. I really have very few comments and found the work well done and well presented. I also hope that in the future the authors consider how other types of choices such as between rewards of similar value but of different preference, reward values, etc. may have similar or different underlying mechanisms, especially given the correlation of drift rate observed.

1) The main distinction was that it was hard to understand the benefits of the ExGauss vs. DDM approach. They capture different aspects of behavior and I felt that figure 2 could potentially lay out more what one would "get" by using one approach or the other.

2) The biggest downside to the work is that it is exclusively in male rats which is not discussed currently. The authors should discuss how they may expect the work to be improved or not by the addition of female subjects and the known differences in decision making across males and females (for example work from Nicola Grissom).

3) I may have missed it but does the correlation between drift rate and high value choice develop or change across training?

Reviewer 2

Major:

1) Figure 6. Can the authors provide an interpretation for the differences observed with HDDM and PyDDM models on drift rate and non-decision time parameters.

2) Line 368-369. The authors propose that the ACC is a candidate region that may undergo changes during early learning. It would be useful to expound a bit more on this point.

3) Given the relatively rapid evolution of decision-making process in this task over a few sessions, have the authors considered examining how decision making parameters change within the first session itself, for example by comparing trials in the first and second halves of session 1?

Minor:

1. Line 137, capitalize 'Single'

2. Line 387, should the figure callout be 4C here?

## Author Response

Response to Reviews We thank the editor and reviewers for their positive comments on our study. We have revised the paper to address the minor concerns that were raised. We hope that the paper is now acceptable for publication in eNeuro.

Comments from each reviewer are listed below, with our responses in blue and new text in the manuscript in blue italics. Changes in the manuscript have been highlighted using "track changes" editing, and provided as a PDF for the review of the changes. Figures and legends have been removed from being in-line and are included at the end of the clean manuscript file.

Reviewer 1 The main distinction was that it was hard to understand the benefits of the ExGauss vs. DDM approach.

They capture different aspects of behavior and I felt that figure 2 could potentially lay out more what one would "get" by using one approach or the other.

We very much appreciate this suggestion. Our goal of Figure 2 was to make sure that any reader understood what each type of model was based on, and not to relate them to one another. Both types of models account for distinct global measures of performance and are not trial-by-trial measures. As such, it is not entirely clear how to graphically relate them to each other. We added text to the manuscript, in the Methods section, to to clarity what the parameters of each type of model represent in terms of behavioral performance). We restructured the paragraph headings in that part of the manuscript and added the text below. We hope that this change is acceptable.

New text:

Data Analysis: Computational Models Two different types of computational models were used to understand how early choice learning affected the rats' performance in the decision-making task (Fig 2). ExGauss models were used to account for the statistics of response times from different kinds of trials. Drift Diffusion models were used to account for the dynamics of the decision process over the period of initial choice learning.

The biggest downside to the work is that it is exclusively in male rats which is not discussed currently.

The authors should discuss how they may expect the work to be improved or not by the addition of female subjects and the known differences in decision making across males and females (for example work from Nicola Grissom).

This is an excellent point. As reported in the original version of the paper, these studies were initiated before the NIH implemented their policy for including equal numbers of male and female subjects in animal studies. However, it is well established that there are sex differences in decision-making performance, as highlighted by the work of Nicola Grissom and colleagues. In the context of the present study, we have since ran a new study with an equal number of female and male rats, using the same behavioral task as reported here (Palmer et al., 2024). We found a clear effect of sex as a factor.

Specifically, the female rats showed higher preferences for the larger reward and higher drift rates based on DDM models. This study was recently preprinted and is presently under review at the Journal of Neuroscience.

We added a paragraph to the Discussion to address this issue, citing both work by Dr Grissom's team (Chen et al., 2021) and our own follow-up to the present study. The text is below. We also revised the Methods section, where we had previously commented on the rationale for the study only having male rats, as that text is now redundant with the new text in the Discussion.

Limitations A major limitation of the present study is that only male rats were used. The experiments were initiated prior to the implementation of the NIH policy that studies should use equal numbers of male and female animals.

However, since that time, evidence has been found for effects of sex on decision making in rodents (Chen et al., 2021). Another recent study from our laboratory used the same behavioral task as the present study and included equal numbers of female and male rats (Palmer et al., 2024). Female rats showed higher preferences for stimuli associated with the high-concentration liquid sucrose rewards and higher drift rates from DDM modeling compared to male rats. Male rats showed more dramatic effects of initial choice learning compared to females. Based on this follow-up study, it is possible, if not likely, that the results reported in the present paper are relevant for choice learning by male, but not female, rats.

I may have missed it but does the correlation between drift rate and high value choice develop or change across training? Drift rate was generally stable across learning. The HDDM models found that it was lower in the first session of choice learning compared to the last session of choice learning, but not compared to other learning sessions (Left plots in Fig 6A). The PyDDM models found no evidence for a change in drift rate over sessions (Right plot in Fig 6A). Likewise, high value choice was stable over days (Fig 4C). Repeated measures correlation found a significant correlation between the two measures (Fig 7A), and no effects of rat or session on the strength of the correlation. Therefore, there was no evidence that the relationship between these measures changed with learning. We added a sentence to the paragraph describing Fig 7A, shown below:

We used repeated-measures correlation to assess how the behavioral and computational measures reported above related to each other. For this analysis, we used the parameters from the PyDDM models and related them to the animals' preferences for the higher value stimulus and measures of their response times based on ExGauss modeling. The strongest correlation across measures was between the drift rate and the percent of trials in which the rats chose the higher value stimulus (r=0.9222, df=69, p<0.001, CI95%: 0.88-0.95) (Fig 7A). The repeated-measures correlation analysis did not find evidence for effects of rat or session on the strength of this association.

Reviewer 2 Figure 6. Can the authors provide an interpretation for the differences observed with HDDM and PyDDM models on drift rate and non-decision time parameters.

This is a good question. For the drift rate, the overall patterns over sessions were the same for the two DDM models. The values from the PyDDM models were not sensitive to training session. The parameters from the HDDM models were only different for the first and fifth training session. Those sessions did not differ from the other sessions, e.g. drift rate was not different between sessions two, three, or four. The same was true for non-decision time, the only difference found by HDDM was between the first and fifth training sessions.

One possible reason for the increased sensitivity of the HDDM models is that they were generated in a hierarchical manner, based on 2500 models. By contrast, the PyDDM models were based on a single model for each rat. We added a comment to the Results to offer this potential explanation of the differences between the two kinds of DDM models.

In contrast to the results from the HDDM models, the PyDMM models did not find differences in the drift rate and non-decision time parameters for the first and fifth training sessions. One possible reason for the increased sensitivity of the HDDM models is that they were generated in a hierarchical manner, based on 2500 models. By contrast, the PyDDM models were based on a single model for each rat.

Line 368-369. The authors propose that the ACC is a candidate region that may undergo changes during early learning. It would be useful to expound a bit more on this point.

The role of the ACC (at least a part of the rodent ACC, the prelimbic area) is addressed in a follow-up study to the present paper (Palmer et al., 2024). It shows that reversible inactivation of the prelimbic cortex reduces the decision threshold, in the same task used in the present study. We added a citation to this study, as stated in the next text below.

This region of the rat brain has been shown to be crucial for the maintenance of the decision threshold in a recent study (Palmer et al., 2024) that used the same task as in the present study.

Given the relatively rapid evolution of decision-making process in this task over a few sessions, have the authors considered examining how decision making parameters change within the first session itself, for example by comparing trials in the first and second halves of session 1? We did consider this important issue. However, we did not report on within-session effects of learning using DDM modeling for several reasons. First, while our rats performed ~300 trials per session, only about 100 of these trials were dual-offer trials. Running models for only 50 trials seems to be really pushing the limits on fitting the DDM parameters. Second, as shown in Fig 2D, the rats exhibited slower responses and longer inter-trial intervals later in the testing sessions. By contrast, trials early in the session were performed with shorter overall response times and at a quicker pace. These differences makes it hard to ensure that results of DDM fits to the first and second halves of the session would be interpretable. For these reasons, we did not use DDM models to evaluate within-session effects of learning.

Also, the two minor issues (capitalization of Single on line 137 and the citation of Figure 4C on line 387 were fixed, along with some other minor type errors that were found in finalizing this version of the paper.
