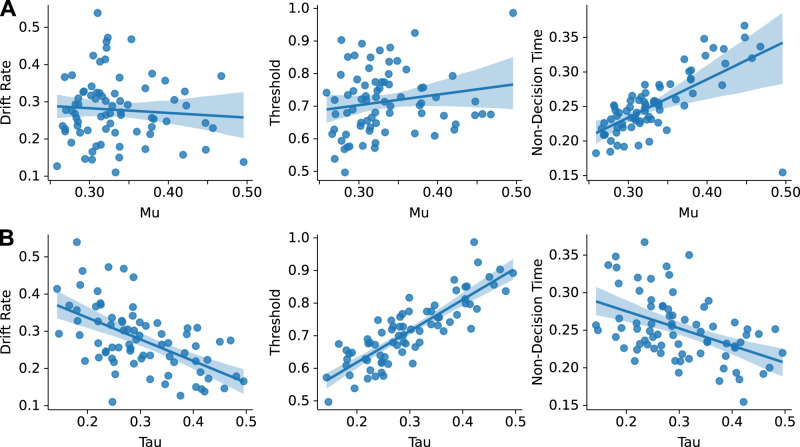# Erratum: White et al., “Learning to Choose: Behavioral Dynamics Underlying the Initial Acquisition of Decision-Making”

**DOI:** 10.1523/ENEURO.0429-24.2024

**Published:** 2024-11-05

In the article “Learning to Choose: Behavioral Dynamics Underlying the Initial Acquisition of Decision-Making,” by Samantha R. White, Michael W. Preston, Kyra Swanson, and Mark Laubach which was published online on May 9, 2024, the final edits to the accepted article were not incorporated into the published article. The text has been updated to reflect the changes requested at peer review.

In addition, the right graph in Figure 8*A* was rendered incorrectly due to a production error.

The changes do not affect the conclusions of the paper, and the online version has been corrected.

**Figure 8.**